# TNFα is a trigger of aging-associated liver inflammation in mice

**DOI:** 10.1038/s41514-025-00326-w

**Published:** 2026-01-13

**Authors:** Haktan Övül Bozkir, Annette Brandt, Katja Csarmann, Anja Baumann, Katharina Burger, Timur Yergaliyev, Tim Hendrikx, Amélia Camarinha-Silva, Ina Bergheim

**Affiliations:** 1 Department of Nutritional Sciences, Molecular Nutritional Science, University of Vienna, Vienna, Austria; 2 Institute of Animal Science, University of Hohenheim, Stuttgart, Germany; 3 Department of Laboratory Medicine, KILM, Medical University of Vienna, Vienna, Austria

**Keywords:** Diseases, Gastroenterology, Immunology, Medical research

## Abstract

Tumor necrosis factor α (TNFα) regulates inflammation in metabolic diseases and probably aging-associated inflammation. Here, TNFα´s role in aging-related liver inflammation and fibrosis and underlying mechanisms was assessed in mice. In male C57BL/6J mice, aging increased hepatic inflammation, senescence markers *p16* and *p21* and *Tnfa* mRNA expression in liver tissue. In a second study, 4 and 24-month-old TNFα^-/-^ and wild-type (WT) mice were compared for senescence, liver damage, intestinal barrier function, and microbiota composition. 24-month-old TNFα^-/-^ mice were significantly protected from the aging-associated increase in hepatic senescence, inflammation and fibrosis found in WT mice. This protection was related with preserved stem cell marker expression, maintained small intestinal barrier function and lower bacterial endotoxin in portal blood. While differing from young mice, intestinal microbiota composition of old TNFα^-/-^ mice differed markedly from age-matched WT mice. Also, TNFα was found to alter permeability and tight junction protein levels being reversed by the presence of an JNK inhibitor in an ex vivo intestinal tissue model. Taken together, our results suggest that TNFα plays a key role in the development of aging-related liver decline in male mice.

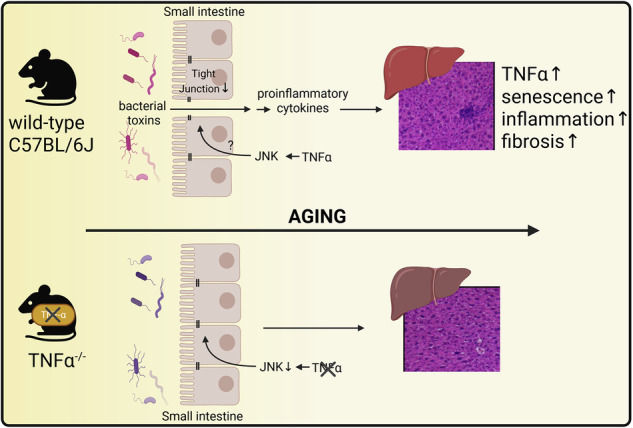

## Introduction

In 2022, it has been projected by the WHO that the number of individuals aged 60 years and older will outnumber the number of children below the age of 15 within the next ~25 years^1^. At the same time, it has been suggested that the general life-span is increasing, especially in well-developed countries; however, it has also been proposed that healthy life expectancy will not increase to the same extent as the time spent in suboptimal health^2^. Indeed, it has been shown that older age is an individual risk factor for the development of several non-communicable diseases including Alzheimer´s disease, type 2 diabetes and metabolic dysfunction-associated liver disease (MASLD), previously known as non-alcoholic fatty liver disease (NAFLD), referring to a spectrum of liver disorders associated with metabolic dysfunction, encompassing simple steatosis, non-alcoholic steatohepatitis (NASH), and related fibrosis^3–6^. Even in so called “healthy aging” it has been shown that aging is associated with a degeneration of cells, tissues, and even whole organs^7^. Studies also suggest that even in the absence of any overt infections or injuries, aging is afflicted with a low-grade inflammation having led to the term “inflammaging”, a phenomena of chronic, low-grade, systemic inflammation^8,9^. Aging-related decline of the liver in settings of ´healthy aging´ has been related to so called hallmarks of aging, including mitochondrial dysfunction, epigenetic alterations and telomer attrition, and changes of intestinal microbiota and intestinal barrier dysfunction^10–12^.

A misregulation of the expression of the proinflammatory cytokine tumor necrosis factor α (TNFα) has been linked to the development of many diseases, including type 2 diabetes and MASLD (for overview see refs. ^13,14^). Studies in elderly humans and rodents suggest that, besides displaying elevated TNFα protein levels in blood, older age is also associated with increased levels of TNFα in liver tissue^15–17^. Studies in TNFα^-/-^ mice suggest that TNFα, through c-jun N-terminal kinases (JNK)-dependent signaling cascades, can act as a regulator of other cytokines like interleukin 1β (IL1β) and IL6 in the liver, thereby diminishing the development of liver diseases^18^. In the same study, it was shown that a genetic deletion of TNFα diminished the bacterial endotoxin-dependent induction of IL1β in immune cells^18^. The critical role of TNFα as a key regulator of the onset of hepatic inflammation and thereby the development of liver damage has been supported by studies employing anti-TNFα antibodies^18–20^. Indeed, it was shown that a treatment with anti-TNFα antibodies not only improved liver histology but also was related to a reduction of other proinflammatory cytokines^18,21^. However, if TNFα is contributing to aging-related liver degeneration has not yet been clarified.

Hence, the aim of the present study was to determine the expression of TNFα in the natural course of aging and investigate if a loss of TNFα attenuates aging-related changes in the liver, e.g., the development of inflammation and fibrosis in mice.

## Results

### Markers of inflammation and liver damage in aging C57BL/6J mice

**Fig. 1:**
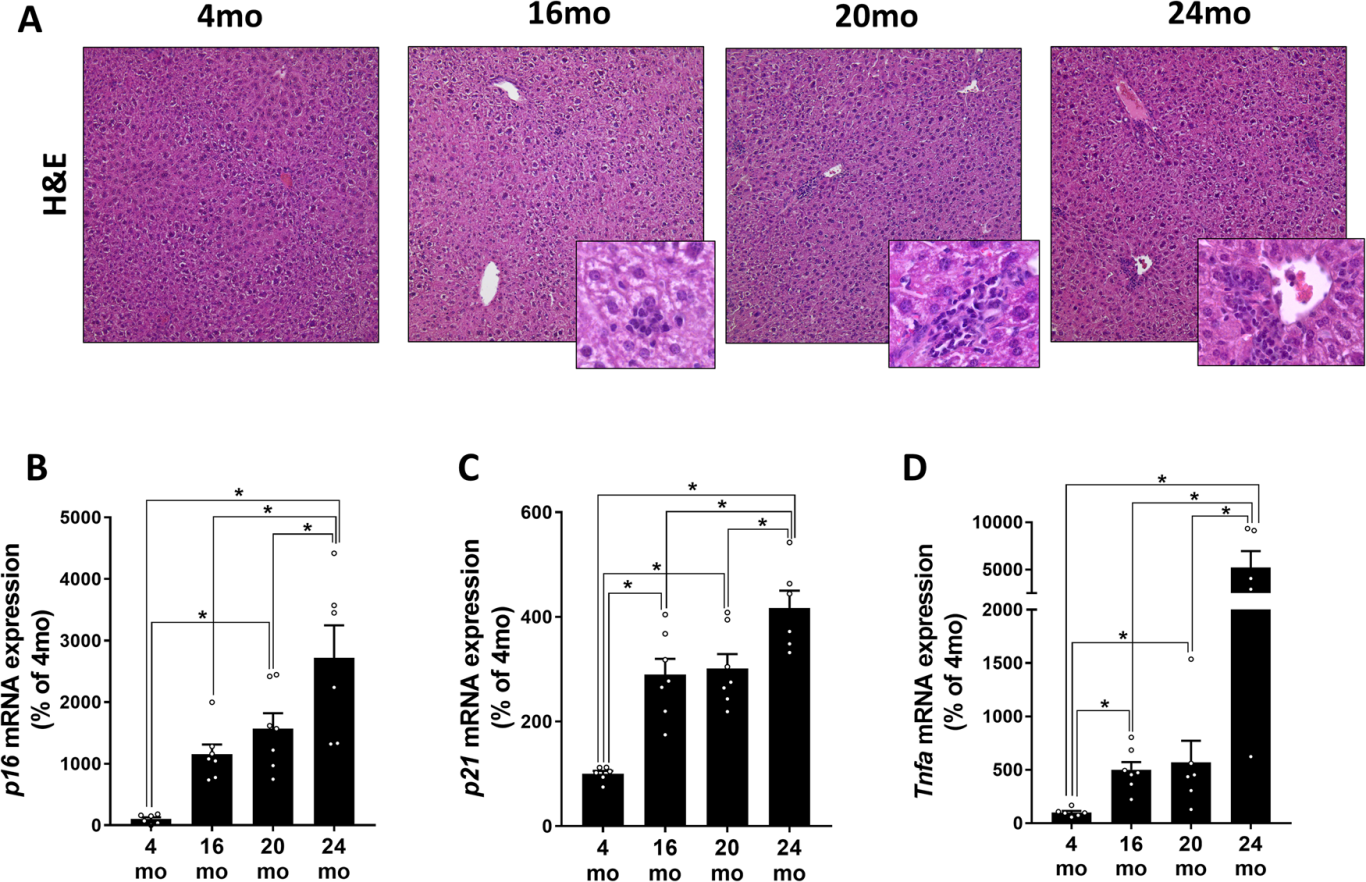
Markers of inflammation and liver damage and TNFα expression in livers of aging male C57BL/6J mice. **A** Representative pictures of haematoxylin & eosin (H&E) stained liver sections (100x, 400x), mRNA expression of (**B**) *p16*, (**C**) *p21* and (**D**) *tumor necrosis factor α* (*Tnfa*) in livers of 4-month-old (mo), 16mo, 20mo, and 24mo old male wild-type C57BL/6J mice. Data are shown as means ± SEM. *n* = 5–7. *p < 0.05. *p*-values were determined by **B**–**D**: one-way ANOVA followed by Tukey´s Post Hoc test.

In line with previous findings^10,15^ aging in male C57BL/6J mice was related with the development of inflammatory foci and an increased mRNA expression of *p16* and *p21* as well as of *Tnfa* in liver tissue of mice (Fig. 1A–D). As differences with respect to markers of senescence but also TNFα between age groups were most pronounced when comparing 4 and 24-month-old mice, all further studies were carried out in mice aged 4 and 24 months.

### Markers of inflammation and liver damage in TNFα^-/-^ and wild-type C57BL/6J mice

**Fig. 2:**
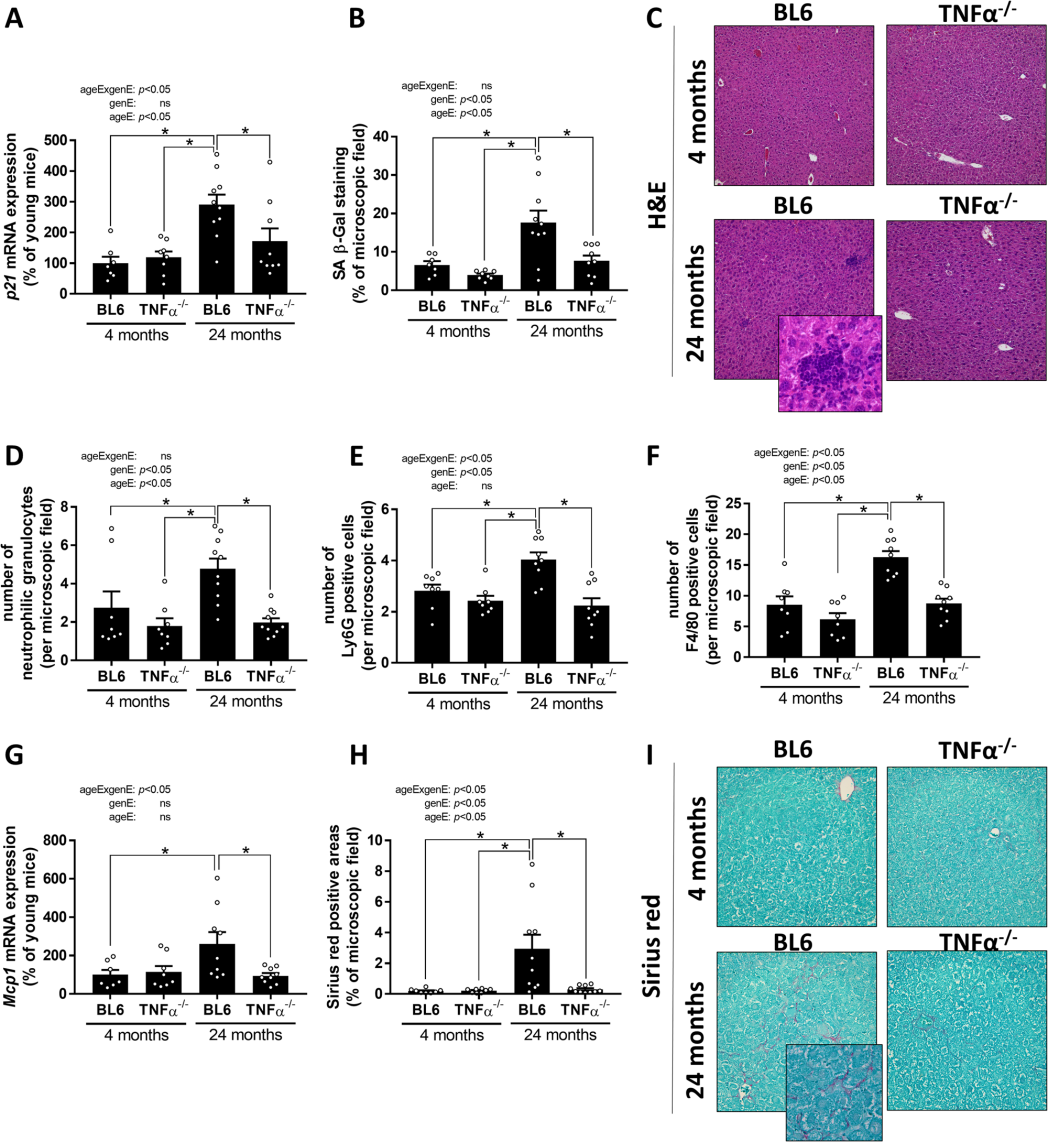
Indices of liver inflammation and fibrosis in young and 24 months old C57BL/6J and TNFα^-/-^ mice. **A** *p21* mRNA expression in hepatic tissue, **B** senescence-associated β-galactosidase (SA β-Gal) staining in liver sections, and **C** representative pictures of H&E staining (100x and 400x), number of (**D**) neutrophilic granulocytes, and (**E**) lymphocyte antigen 6 complex locus G6D (Ly6G) positive cells per microscopic field. **F** Number of F4/80 positive cells, **G** *monocyte chemoattractant protein-1* (*Mcp1*) mRNA expression in liver and **H** densitometric analysis of sirius red positive areas per microscopic field as well as (**I**) representative pictures of sirius red staining (200x and 400x) in livers of young (4 months) and old (24 months) wild-type C57BL/6J (BL6) and TNFα^-/-^ mice. Data are presented means ± SEM. ageE age effect, genE genotype effect, ageExgenE interaction between age and genotype. **A**, **B,** **D**–**H**: 4-month-old mice: *n* = 7–8, 24-month-old mice: *n* = 9–10. *p < 0.05. *p*-values were determined by **A**, **B**, **D**–**H**: two-way ANOVA followed by Tukey´s Post Hoc test.

In line with previous studies^10,15^, markers of senescence, hepatic inflammation, and fibrosis were significantly higher in 24-month-old wild-type mice than in 4-month-old wild-type and TNFα^-/-^ mice (Fig. 2A–I). In contrast, similar differences between age groups were not found when comparing 24-month-old TNFα^-/-^ mice with 4-month-old wild-type and TNFα^-/-^ mice. Rather, markers of senescence, such as *p21* mRNA and senescence-associated β-galactosidase staining (SA β-Gal), numbers of neutrophils (as assessed by Naphthol AS-D chloroacetate esterase staining and lymphocyte antigen 6 complex locus G6D (Ly6G)-antibody staining) and F4/80 positive cells as well as *monocyte chemoattractant protein-1* (*Mcp1*) mRNA expression in 24-month-old TNFα^-/-^ mice, respectively were almost at the level of that found in livers of young mice and significantly lower than in livers of 24-month-old wild-type mice. *Cd11b* mRNA expression tended to be lower in livers of aged TNFα^-/-^ mice when compared with age-matched wild-type mice (*p* = *0.064*, not statistically significant) (Fig. 2A–G, Table 1, Supplementary Fig. 1). Neither wild-type nor TNFα^-/-^ mice showed any signs of hepatic steatosis (Fig. 2C). Sirius red-stained areas were also significantly lower in livers of 24-month-old TNFα^-/-^ mice compared to age-matched wild-type mice (Fig. 2H, I). However, neither *α smooth muscle actin* (*asma)* nor *collagen type I α 1 chain (col1a1)* or *matrix metalloproteinase-2 (Mmp2)* and *tissue inhibitor of metalloproteinases 2 (Timp2)* mRNA expression in liver tissue differed between groups (Table 1). Also, activities of aspartate aminotransferase (AST) and alanine aminotransferase (ALT) in plasma were similar between groups (Table 1). As there were no signs of liver damage observed in young C57BL/6J mice or young TNFα^-/-^ mice, young C57BL/6J were used to be representative young controls in all further assessments.

**Table 1:** Body weight and markers of liver damage in wild-type C57BL/6J  and TNFα^-/-^ mice

Parameter	Groups			
	4 months		24 months	
	BL6	TNFα^-/-^	BL6	TNFα^-/-^
Body end weight (g)	32.7 ± 0.9	31.1 ± 0.8	34.2 ± 0.5^b^	33.2 ± 0.5
ALT (U/L)	33.4 ± 4.1	30.5 ± 3.3	35.8 ± 1.4	37.0 ± 2.0
AST (U/L)	42.3 ± 2.3	43.6 ± 2.3	48.9 ± 2.4	49.2 ± 1.4
*Cd11b* mRNA expression^c^ (% of young BL6 mice)	100 ± 24	137 ± 50	497 ± 123 ^a,b^	164 ± 37
*Mmp2* mRNA expression^c^ (% of young BL6 mice)	100 ± 13	92.5 ± 16	135 ± 22	153 ± 32
*Timp2* mRNA expression^c^ (% of young BL6 mice)	100 ± 20	149 ± 28	104 ± 16	97.3 ± 20
*asma* mRNA expression^c^ (% of young BL6 mice)	100 ± 19	133 ± 19	154 ± 35	77.6 ± 9.8
*Col1a1* mRNA expression^c^ (% of young BL6 mice)	100 ± 18	94.5 ± 16	126 ± 19	82.7 ± 14

Values are means ± standard error of means.

*asma* α smooth muscle actin, *AST* aspartate aminotransferase, *ALT* alanine aminotransferase, *BL6* wild-type C57BL/6J, *Col1a1* collagen type I α 1 chain, *Mmp2* matrix metalloproteinase-2, *Timp2* tissue inhibitor of metalloproteinases 2.4 months old mice: *n* = 7–8, 24 months old mice: *n* = 8–10.

*p*-values were determined by two-way ANOVA followed by Tukey´s Post Hoc test.

^a^*p* < 0.05 compared to 4-month-old BL6 mice.

^b^*p* < 0.05 compared to 4-month-old TNFα^-/-^ mice.

^**c**^liver tissue.

### Markers of intestinal barrier function and intestinal stem cell markers in old aged TNFα^-/-^ and wild-type C57BL/6J mice

**Fig. 3:**
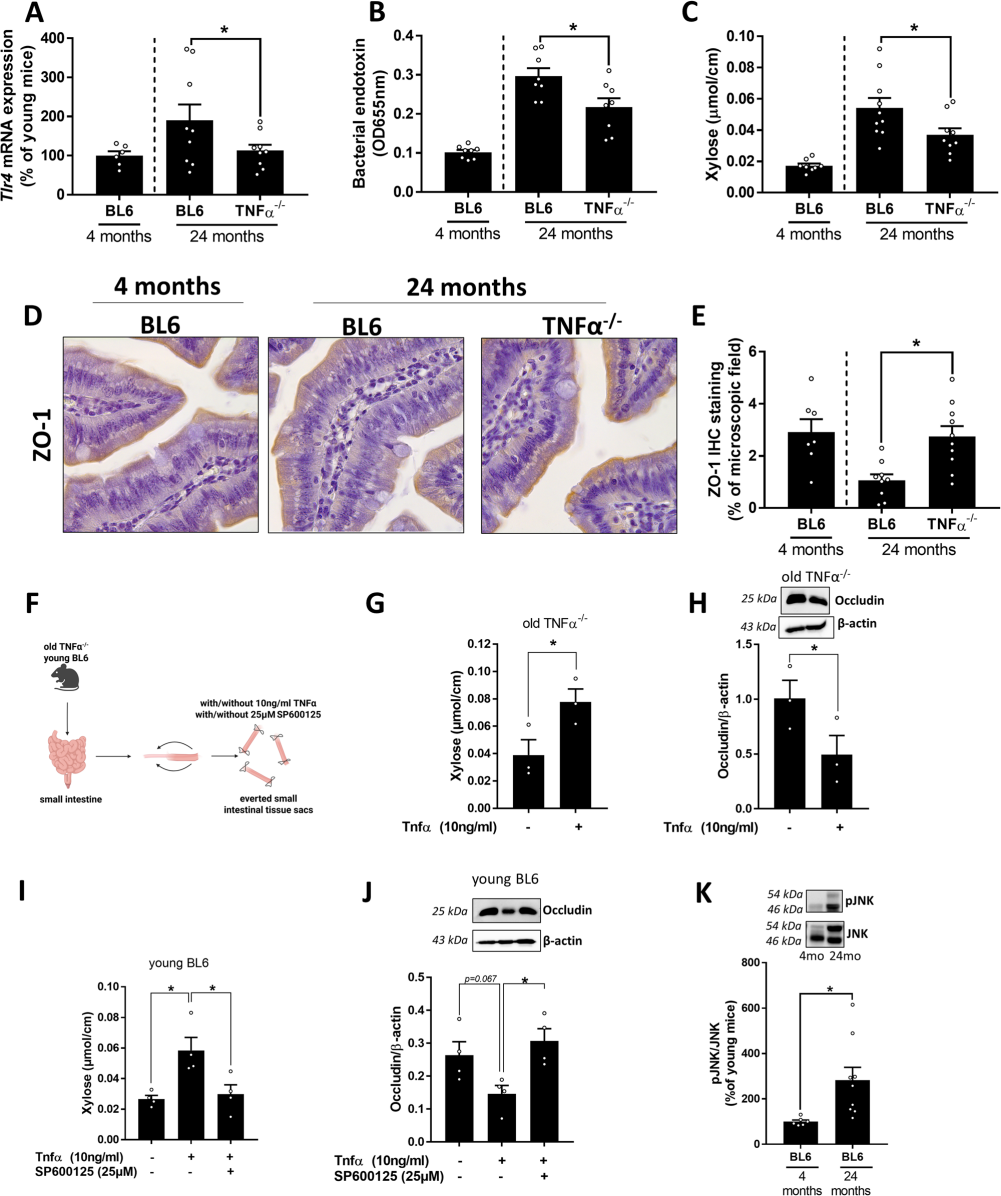
Markers of intestinal barrier function in 24 months old C57BL/6J and TNFα^-/-^ mice. **A** *Toll-like receptor 4* (*Tlr4)* mRNA expression in liver tissue, **B** bacterial endotoxin in plasma, **C** xylose permeation in small intestine determined ex vivo in everted gut sacs **D** representative pictures (630x) and **E** densitometric evaluation of zonula occludens-1 (ZO-1) staining in small intestine of young (4 months) and old (24 months) C57BL/6J (BL6) and TNFα^-/-^ mice. **F** Schematic figure of preparation of ex vivo everted gut sacs. Figure created with Biorender.com. **G** Xylose permeation, **H** representative western blots and densitometric evaluation of Occludin protein concentration of ex vivo everted gut sacs from old TNFα^-/-^ mice (24 months of age) incubated with or without 10 ng/ml TNFα. **I** Xylose permeation, **J** representative western blots and densitometric evaluation of Occludin protein concentration of ex vivo everted gut sacs of young (4 months) BL6 mice incubated with or without 10 ng/ml TNFα and 25 µM c-jun N-terminal kinases (JNK) inhibitor SP600125. **K** Representative western blots and densitometric evaluation of pJNK/JNK protein concentration in small intestinal tissue of young and old wild-type C57BL/6J (BL6) mice. Data are presented means ± SEM. **A**–**E**, **K** 4-month-old mice (shown for comparison): *n* = 6–8, 24-month-old mice: *n* = 8–10, **G**, **H**: *n* = 3, **I**, **J** *n* = 4. *p < 0.05. *p*-values were determined by **A**–**H**, **K** unpaired t-test, **I**, **J** one-way ANOVA followed by Tukey´s Post Hoc test.

As it has been suggested before in various disease settings that TNFα is critical in the regulation of intestinal barrier function, and aging has been shown to be related with intestinal barrier dysfunction^10,11,22^, we next determined *toll-like receptor 4 (Tlr4)* mRNA expression and bacterial endotoxin levels in old aged TNFα^-/-^ mice and age-matched wild-type animals. Both mRNA expression of *Tlr4* in liver tissue and bacterial endotoxin levels in portal blood were significantly higher in wild-type mice than in old-aged TNFα^-/-^ mice (Fig. 3A, B). In line with the findings for bacterial endotoxin, small intestinal permeability as determined ex vivo using xylose as a marker was also significantly lower in old aged TNFα^-/-^ mice compared to age-matched wild-type mice, while intestinal permeation of xylose did not differ between age groups in large intestinal tissue everted sacs (Fig. 3C, Table 2). Also, while gross morphology and villi length as well as width were similar between wild-type and TNFα^-/-^ mice, crypt depth was significantly lower in wild-type mice than in age-matched TNFα^-/-^ mice (Table 2). Furthermore, protein concentration of zonula occludens-1 (ZO-1) in the small intestine was significantly higher in TNFα^-/-^ mice compared to old wild-type mice, being almost at the level of young C57BL/6J mice (Fig. 3D, E). The abolished loss of tight junction proteins in the small intestine was associated with significantly higher mRNA expression of stem cell markers, such as *leucine-rich repeat-containing G-protein coupled receptor 5* (*Lgr5*) and *telomerase reverse transcriptase* (*Tert*) in 24-month-old TNFα^-/-^ mice compared to age-matched C57BL/6 J mice (Table 2). In contrast, mRNA expression of *Cd1d* and *Nk1.1,* both being markers of iNKT cells, a cell type suggested to be altered by aging and to be modulated by TNFα^23,24^, did not differ between both 24-month-old age groups (Table 2).

**Table 2:** Intestinal morphology and intestinal stem cell markers in wild-type C57BL/6J and TNFα^-/-^ mice

Parameter	Groups		
	4 months	24 months	
	BL6^b^	BL6	TNFα^-/-^
Xylose large intestine (µmol/cm)	0.023 ± 0.003	0.019 ± 0.003	0.017 ± 0.001
Villi length (µm)	259 ± 7.8	285 ± 9.2	300 ± 19
Crypt depth (µm)	114 ± 8.5	101 ± 8.7	131 ± 13^c^
Villi width (µm)	77.2 ± 1.2	92.4 ± 2.7	100 ± 7.3
*Tert* mRNA expression^a^ (% of young BL6 mice)	100 ± 21	66.5 ± 6.1	99.7 ± 11^c^
*Lgr5* mRNA expression^a^ (% of young BL6 mice)	100 ± 28	74.0 ± 7.7	122 ± 25^c^
*Cd1d* mRNA expression^a^ (% of young BL6 mice)	100 ± 26	116 ± 18	110 ± 17
*Nk1.1* mRNA expression^a^ (% of young BL6 mice)	100 ± 38	116 ± 21	95.8 ± 13

Values are means ± standard error of means. 4-month-old mice: *n* = 6–8, 24-month-old mice: *n* = 8–10 *p*-values were determined by unpaired t-test.

*BL6* wild-type C57BL/6J, *Lgr5* leucine-rich repeat-containing G-protein coupled receptor 5, *Tert* telomerase reverse transcriptase.

^**a**^Small intestinal tissue.

^b^Shown for comparison.

^**c**^*p* < 0.05 compared to 24 months old BL6.

### Effect of TNFα and a JNK inhibitor on intestinal barrier function in ex vivo everted gut sac experiments

To further determine if TNFα may have direct effects on intestinal barrier function in aging mice, intestinal tissue obtained from old TNFα^-/-^ mice was used to build everted gut sacs. These sacs were then challenged with TNFα or vehicle (Fig. 3F). Compared with vehicle treated sacs, permeability, as determined by xylose permeation, was significantly higher, and occludin protein concentration was significantly lower when tissue sacs of the same mice were challenged with TNFα (Fig. 3G, H). In line with these findings, when tissue sacs from young C57BL/6J mice were challenged with TNFα, xylose permeation was also significantly increased. This effect of TNFα was almost completely abolished when tissue sacs exposed to TNFα were concomitantly treated with the JNK inhibitor SP600125 (Fig. 3I). While not reaching the levels of significance, occludin protein concentration was by trend lower in TNFα-treated tissue sacs (*p* = 0.067 compared to untreated sacs), an effect abolished when tissue sacs were concomitantly treated with the JNK inhibitor (*p* < 0.05 vs TNFα-treated sacs). In this tissue, sacs occludin protein concentration was comparable to untreated sacs (Fig. 3J). Furthermore, in the small intestinal tissue of 24-month-old C57BL/6J mice concentration of pJNK was significantly higher compared to young wild-type (C57BL/6J) mice (Fig. 3K).

### Intestinal microbiota composition in old aged TNFα^-/-^ and wild-type C57BL/6J mice

**Fig. 4:**
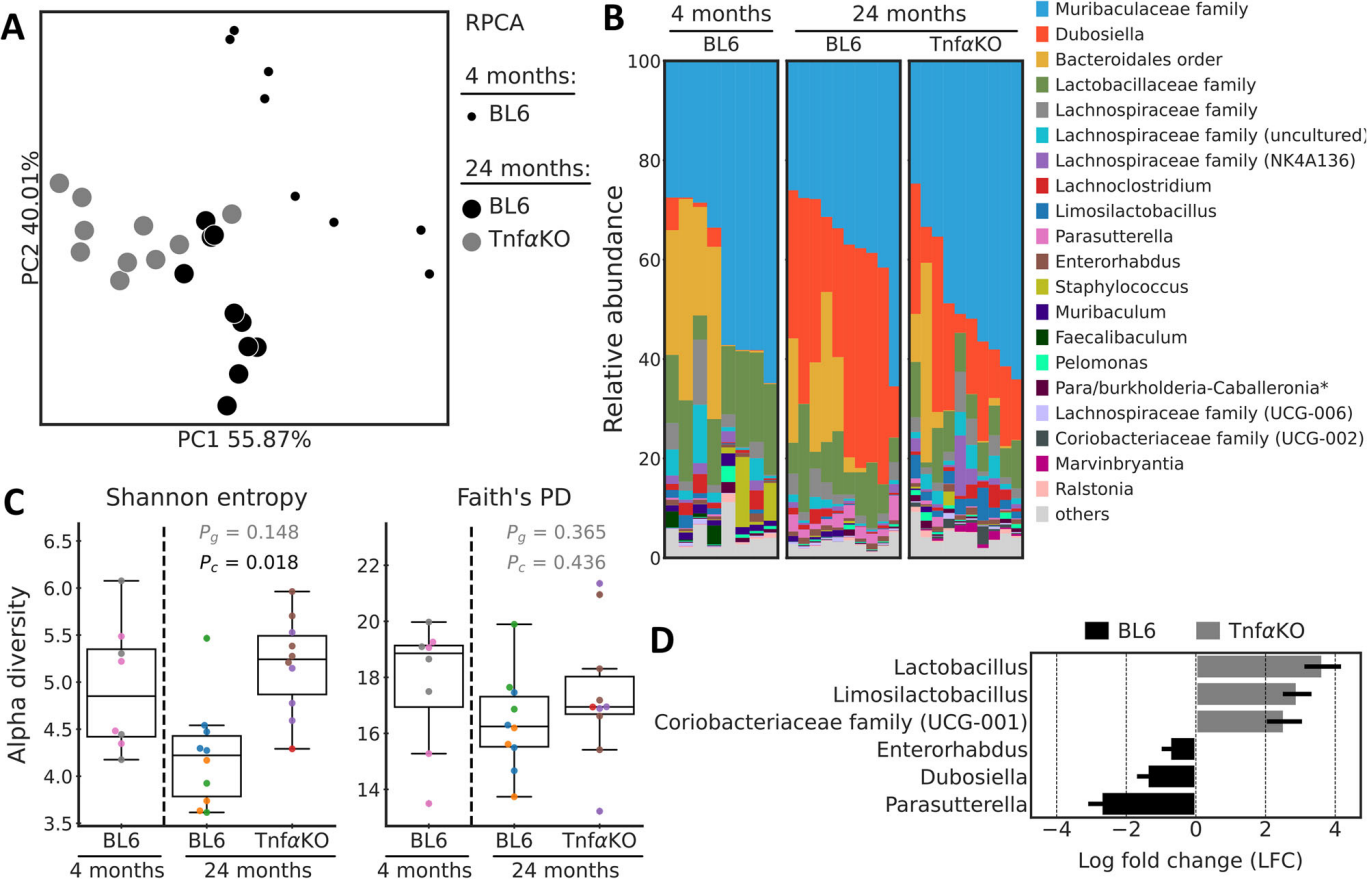
Microbiota diversity, abundance and composition in small intestine of 24 months old C57BL/6 J and TNFα^-/-^ mice. **A** PCoA plots based on RPCA. The marker’s size differentiates age. **B** Taxonomy area plots at the genus level (Silva 138.1). If a genus level was not assigned, the last available taxonomy rank was used for the label. **C** Alpha diversity based on Shannon entropy and Faith’s phylogenetic diversity indices. Dots indicate individual samples and are colored by the cage. **D** Differentially abundant (Ancom-BC) genera between 24-month-old TNFα^-/-^ mice and wild-type C57BL/6 J (BL6) mice. Colors indicate in which group a given genus or taxonomy unit was more abundant compared to other group. *Burkholderia-Caballeronia-Paraburkholderia.

To investigate whether the genetic deletion of TNFα affected intestinal microbiota, the composition was assessed in the small intestine. Microbiota composition was significantly different between 24-month-old TNFα^-/-^ mice and age-matched wild-type mice. The Adonis test, performed on the RPCA distances (Fig. 4A), revealed differences between TNFα^-/-^ mice and age-matched wild-type mice with respect to their beta diversity (*p* = 0.001 after correction for cage effects). Regarding the alpha diversity (Fig. 4C), no differences between both age groups on Faith’s PD (*p* = 0.365) and Shannon entropy (*p* = 0.148) were found.

Across the study, in all groups unclassified to the genus level members of the *Muribaculaceae* family dominated the bacterial community (Fig. 4B). Relative abundances of *Bacteroidales* varied across the animals and groups. *Dubosiella*, poorly represented in 4-month-old mice, was the second most abundant genus in 24-month-old mice independently from the genotype. When tested with Ancom-BC (Fig. 4D) for differential abundance, the *Lactobacillus*, *Limosilactobacillus,* and members of the *Coriobacteriaceae* family (UCG-001) were more abundant in the 24-month-old TNFα^-/-^ mice compared to age-matched wild-type mice. At the same time, the abundance of *Enterorhabdus*, *Dubosiella,* and *Parasutterella* genera was higher in the aged BL6 mice compared to TNFα^-/-^ mice.

## Discussion

Aging is a significant risk factor for the development and progression of various liver diseases, including MASLD, alcohol-associated liver disease (ALD), and viral hepatitis^25^. Notably, even in the absence of additional risk factors such as an unhealthy diet or consumption of large amounts of alcohol, the development of inflammation and fibrosis is related to aging^15,26,27^. In the present study, the development and progression of inflammatory alterations and senescence in the liver tissue of male mice paralleled the increase of *Tnfa* mRNA expression in the liver tissue. Furthermore, in old-aged TNFα^-/-^ mice, signs of inflammation, and fibrosis, but also of senescence in liver tissue were markedly lower than in age-matched wild-type mice. Results of our studies are somewhat in line with studies of others in humans, reporting that aging is associated with an increase in circulating TNFα and that every 10-year increase in age was related with a 23% increase in TNFα production normalized to leukocytes, even in the absence of a stimulus^28,29^. Studies have shown before that TNFα-deficient mice are protected from aging-associated inflammatory alteration in the lung^30^ and that at a loss of TNFα or its receptor TNFR1 can attenuate the development of inflammation in dietary models of MASLD but also ALD^18,31,32^. Indeed, TNFα is considered a central mediator of chronic inflammatory diseases and has been suggested to induce senescence^33–35^. However, its role in the development of fibrosis remains controversial. In some studies, it has been suggested that an activation of TNFα signaling, particularly through Kupffer cells, is critical for the development of hepatic fibrosis^36^ while in some earlier studies, it has been suggested that TNFα may exert anti-fibrogenic effects^37–39^. In the present study, histological signs of fibrosis like Sirius Red staining, was at the level of young mice in livers of TNFα^-/-^ mice while wild-type mice showed markedly more staining, being indicative of collagen fibers. It has been shown before that aging is related to higher collagen deposition in the liver and an activation of stellate cells^15,40^. In the present study, while somewhat varying especially in the wild-type group, mRNA expression of *asma* and *col1a1* was similar between groups, suggesting stellate cells were not (yet) activated and that maybe other mechanisms might be involved in the onset of aging-related fibrotic alterations. Indeed, besides an enhanced synthesis, a dysbalance in degradation, e.g., resulting from alterations in the balance of MMPs and TIMPs, may also be critical in an enhanced deposition of collagen in the liver^41,42^. It has been shown that the activity of MMPs and particularly MMP-1 and -2 declines with age, while the activity of their inhibitor TIMP1 increases^43,44^. In the present study, neither *Mmp2* nor *Timp2* mRNA expression were affected by age or the genetic deletion of TNFα. These findings are in line with those of others who also found no aging-associated differences in *Mmp2* and *Timp2* mRNA expression levels in liver tissue^45^. Further studies are needed to identify mechanisms underlying the regulatory impact of TNFα in aging liver with respect of fibrosis. Also, despite marked signs of inflammation and fibrosis, ALT and AST activity in plasma was similar between groups. This finding aligns with results from a cross-sectional human study, which suggests that while ALT levels even tend to decrease with age, AST levels remain stable^46^. Although elevated ALT levels are commonly indicative of active hepatic inflammation. Still, patients with chronic liver disease and advancing fibrosis may exhibit normal ALT values^47^. Studies in humans suggest that in MASLD and MASH patients, liver triglyceride content may be the key contributor to the elevation of serum aminotransferase levels. Indeed, the severity of MASH has been shown to be comparable between patients with normal and elevated ALT levels^48^. Contrasting the findings of others reporting that aging in mice is related with the development of steatosis^49^, in the present study, neither wild-type mice nor TNFα^-/-^ mice developed signs of hepatic steatosis. Results of recent studies have shown that the age-related accumulation of fat in the liver is dependent upon the chow fed^50^. Specifically, it was shown that the intake of a refined/purified diet (AIN diet formulas) results in the accumulation of hepatic fat in aging mice, while age-matched mice fed ‘standard´ chow made from natural resources showed no accumulation of fat^50^. In the present study, this kind of ‘standard´ chow was employed. In summary, our data support the hypothesis that TNFα is a critical driver of aging-associated liver inflammation and fibrosis. However, it remains to be determined whether inhibiting TNFα signaling during the course of aging, such as when aging-associated liver degeneration begins to manifest, has beneficial effects on mitigating liver decline and subsequently health span in mice and humans. Also, it remains to be determined if TNFα is also a critical driver of aging in female mice and even more so in humans.

Results from previous studies in human and mice suggest that liver degeneration during aging is associated with altered intestinal microbiota composition and impaired intestinal barrier function in the small and large intestine^10,11,51–53^. Studies also indicate that an increased translocation of bacterial toxins and the induction of TLR-dependent inflammatory signaling cascades in liver tissue may contribute to aging-associated liver decline^10,11,54^ but may also be critical in the induction of TNFα^51^. Moreover, studies have suggested that TNFα is a critical driver of aging-related intestinal dysbiosis and permeability^30^. Somewhat in line with these findings, in the present study, the protection from aging-related liver inflammation and fibrosis found in TNFα^-/-^ mice was related with a protection from aging-associated intestinal barrier dysfunction in small intestinal tissue as determined by xylose permeation and tight junction protein levels, but also bacterial endotoxin levels and *Tlr4* mRNA expression in liver tissue. Interestingly, no difference in xylose permeation between young and old mice was found in the large intestine. It could be that in this part of the gut, intestinal permeability was not (yet) altered or that the marker used was too small to detect a difference^55^. This remains to be determined. In line with the other findings in small intestinal tissue, intestinal morphology, like the decrease in depth of crypts, was less pronounced in TNFα^-/-^ mice compared to age-matched wild-type mice being related with higher expression of stem cells markers in the small intestine. While it still needs to be determined whether aging is associated with small intestinal morphological changes^56,57^, studies suggest that a general stem cell exhaustion and the associated decline in regenerative capacity of the intestinal barrier is related with aging^58^. Moreover, in ex vivo experiments, challenging small intestinal tissue of aged TNFα^-/-^ mice with TNFα resulted in an increase of xylose permeation and loss of tight junction proteins, further suggesting that in aging, similar to the findings in models of colitis and patients with inflammatory bowel disease^59–61^, TNFα may have direct effects on intestinal permeability. Indeed, results of our studies suggest that TNFα may alter intestinal permeability in the small intestine, and JNK inhibitors could reverse these effects. In the present study, the increased permeability of xylose and loss of the tight junction protein occludin was almost completely attenuated in small intestinal tissue sacs which were treated with a JNK inhibitor. Activation of JNK was higher in the small intestinal tissue of old mice than in young wild-type animals. It has also been proposed that the TNFα-dependent regulation of other cytokines and its receptor may be involved in the activation of JNK and related signaling cascades^62–64^. Findings from other studies suggest a regulatory role for JNK in intestinal barrier function in a necrotizing enterocolitis model^65^ and highlight the broader contribution of JNK signaling to age-related pathologies^66^. In line with these findings, in the present study, the JNK inhibitor SP600125 attenuated TNFα-induced increases in intestinal permeability in an ex vivo model of intestinal barrier in young male wild-type mice. It has also been shown that the genetic deletion of TNFα, but also a treatment of old mice with an anti-TNFα antibody, may affect fecal microbiota composition in old but not young mice^30^, being in line with findings of the present study. In the studies of Thevaranjan et al., it was also shown that fecal microbiota composition was not different between young TNFα^-/-^ mice and wild-type mice^30^. In the present study, the age-related changes in microbiota composition observed in wild-type mice were not detected in TNFα^-/-^ mice. However, the microbiota composition of TNFα^-/-^ mice still differed from that of young control mice. Indeed, studies suggest that the intestinal microbiota might play a role as a modulator of healthy aging^46^. Members of the *Coriobacteriaceae* family (UCG-001), which are suggested to decline in midlife^67^ and show reduced abundance in patients of inflammatory bowel disease^68^ were more abundant in TNFα^⁻/⁻^ mice compared to wild-type mice. Additionally, it has recently been proposed that *Lactobacillus* strains, which are more abundant in aged TNFα^⁻/⁻^ mice compared to age-matched wild-type mice, might exhibit potential anti-aging properties^69^. Moreover, studies suggest that intestinal homeostasis and microbiota composition is also regulated by iNKT cell^70^ and that TNFα can also affect iNKT cells function and distribution^24^. In the present study, while intestinal microbiota composition differed between aged wild-type and TNFα^-/-^ mice, markers of intestinal iNKT cells were similar. Further studies are needed to determine if and how TNFα and iNKT cells interact in old-aged intestinal tissue. Whether TNFα directly or through a dependent mechanism affects intestinal microbiota composition remains to be determined. Indeed, in more recent studies, it was shown that JNK-dependent intestinal barrier dysfunction was related to alterations of intestinal microbiota and that the host-microbiome homeostasis seems to also depend on intestinal JNK signaling, and that JNK may be critical in regulating goblet cells differentiation and mucus production as well as intestinal morphology^71,72^. In summary, our findings support the hypothesis that an increased translocation of bacterial toxins may drive aging-associated impairments in intestinal barrier function and contribute to liver degeneration through TNFα-dependent signaling pathways and potentially altered JNK activation.

This study has certain limitations that need to be considered when interpreting the data. Firstly, the experiments were conducted exclusively on male mice, which limits the generalizability of the findings to other sexes and species. Indeed, studies suggest that male and female mice show differences in inflammatory stress mechanisms, with males appearing to display higher basal inflammation and lesser responsiveness to stimuli^73^. Additionally, our experiments are only based on whole-body TNFα knockout mice. Therefore, it remains to be determined whether inhibiting TNFα signaling during the course of aging for instance, when aging-associated liver degeneration first manifests, has beneficial effects on mitigating liver decline. Also, microbiome analysis performed in the present study only focused on determining differences in relative abundance of bacterial composition, while results were not validated at a functional level.

Our study suggests that healthy aging in mice is associated with the development of hepatic inflammation and fibrotic changes and that TNFα might play a central role herein. Additionally, our data further suggest that inhibiting TNFα may mitigate the development of aging-related liver decline through indirect measures, e.g., through alleviating the development of impaired intestinal barrier function and potentially altered JNK activation. Further studies are needed to investigate whether similar alterations occur in aging humans and to explore whether targeting TNFα and/or JNK also affects aging-associated liver damage in humans.

## Methods

### Animal experiments

All experiments were carried out in a specific pathogen-free barrier facility, accredited by the Association for Assessment and Accreditation of Laboratory Animal Care (AAALAC). All animal experiments were approved by the local Institutional Animal Care and Use Committee (BMBWF, 2022-0.527.442). In a **first experiment**, liver tissue was obtained from 4, 16, 20, and 24-month-old male C57BL/6J mice (own bred, Jackson Laboratory, Bar Harbor, USA). In a **second experiment**, male TNFα^-/-^ mice (B6.129S-Tnftm1Gkl/J, Jackson Laboratory, Bar Harbor, USA) and wild-type C57BL/6J mice (BL6, Jackson Laboratory, Bar Harbor, USA) were aged until the age of 4 (n = 8 per group) and 24 months (n = 10 per group). Sample size was calculated with a priori power analysis (GPower, University of Kiel, Germany). Animals were fed standard chow (V1534-300, Ssniff GmbH, Germany) *ad libitum* with free access to tap water at all times in all experiments and separately housed in groups. At the end of the respective experiments, mice were anesthetized with a mixture of ketamine/ xylazine (100 mg ketamine/ kg body weight and 16 mg xylazine/ kg body weight, i.p. injection) and euthanised by cervical dislocation. Blood was collected from the portal vein and liver, and small intestinal tissue were snap-frozen or fixed in neutral-buffered formalin for further analyses. In the second experiment, small pieces of small and large intestinal tissue were used to prepare everted gut sacs to determine intestinal permeability as detailed before^74^ and in part described below. Sacrifice and all following measurements were performed in a randomized group order.

### Ex-vivo everted gut sac model and assessment of intestinal permeability

Small and large intestinal tissue from wild-type (C57BL/6J) and TNFα^-/-^ mice was collected after mice were sacrificed, rapidly rinsed with 1x PBS, and everted as described before^74,75^. In brief, to assess intestinal permeability, pieces of small and large intestinal tissue of mice were everted and filled with 1X Krebs-Henseleit-bicarbonate buffer containing 0.2% bovine serum albumin (KRH) buffer and incubated in 0.1% xylose in KRH buffer for 5 min. After incubation, gut sac content was collected to measure xylose permeation. To determine the effect of TNFα and JNK on intestinal barrier function, in a *first experiment*, small intestinal tissue sacs of 24-month-old TNFα^-/-^ mice were incubated in solutions with or without 10 ng/ml TNFα (Sigma-Aldrich GmbH, Germany) for 55 min followed by an incubation with 0.1% xylose for 5 min. In a *second experiment*, small intestinal tissue sacs of 4-month-old C57BL/6J mice were incubated with or without 10 ng/ml TNFα and with or without 25 µM of the JNK Inhibitor SP600125 (Sigma-Aldrich GmbH, Germany, diluted in DMSO) for 55 min (preincubation phase). Sacs were then incubated in KRH buffers containing 0.1% xylose for another 5 min. Xylose concentration was measured as described before^74,76^ and tissue was snap frozen for further analyses.

### Western blot

Western blot was performed to determine tight junction protein Occludin as well as total JNK and phosphorylated JNK (pJNK) in small intestinal tissue^11^. Briefly, protein was isolated from the small intestinal tissue using a commercially available Trizol reagent (TRItidy G™, AppliChem, Germany) to detect Occludin and by homogenizing intestinal tissue in lysis buffer (1 mol/L HEPES, 1 mol/L50 MgCl2, 2 mol/L KCl, and 1 mol/L dithiothreitol) to obtain cytosolic protein (JNK, pJNK). Polyvinylidene difluoride membranes (Bio-Rad, USA) were incubated with primary antibodies (Occludin: Invitrogen, USA; β-actin: Santa Cruz, USA; JNK, pJNK: Cell Signaling Technology, USA), followed by an incubation with the respective secondary antibodies (Cell Signaling Technology, USA)^77^. Band intensity of the proteins was detected using Clarity Western ECL Substrate (Bio-Rad, USA) and analyzed densitometrically using Image Lab Software Version 6.1.0 (Bio-Rad, USA). Band intensities were normalized to ß-actin or in case of pJNK to JNK.

### Assessment of liver damage and histological evaluation

Paraffin-embedded liver sections (4 µm) of mice were stained with haematoxylin and eosin (H&E) (Sigma-Aldrich GmbH, Germany). A commercially available staining kit was used to stain neutrophil granulocytes in liver sections (Naphthol AS-D chloroacetate esterase kit, Sigma-Aldrich GmbH, Germany), and cells were counted under a microscope (Leica, DM6 B, Leica, Germany) as described before^77^. Lymphocyte antigen 6 complex locus G6D (Ly6G) antibody (ab238132, Abcam, UK) as well as F4/80 antibody (ab6640, Abcam, UK) staining was performed on liver sections using the polyclonal antibody of interest as described detailed before and positive stained cells have been counted^78^. Senescence-associated β-galactosidase (SA β-Gal) was assessed histochemically in frozen liver sections (10 µm) as detailed by others^79^. Sirius red staining was performed in liver sections as described before^80^. Pictures were taken using a camera-attached microscope from randomly selected areas, avoiding the areas rich in blood vessels due to their high-collagen dense nature, and were evaluated densitometrically using Leica Application Suite (LAS 4.5, Leica, Germany) software. Aspartate aminotransferase (AST) and alanine aminotransferase (ALT) levels were measured from mouse plasma in a routine laboratory at the Veterinary Medical University of Vienna, Vienna, Austria.

### Immunohistochemical staining and assessment of intestinal morphology

Zonula occludens-1 (ZO-1) was determined on paraffin-embedded small intestinal tissue Section (4 µm), using the respective primary antibody (anti-ZO-1, Invitrogen, USA), as described detailed before^54^. Additionally, intestinal morphology, e.g., villi width, villi length, and crypt depth in µm was assessed using H&E stained small intestinal tissue Section (4 µm) using analysis software Leica Application Suite X (LASX, Leica, Germany) integrated to microscope (Leica, DM6 B, Leica, Germany).

### Endotoxin levels

Bacterial endotoxin levels in plasma were assessed as previously described, using commercially available SEAP reporter HEK293 cell assay (Invivogen, USA)^54^.

### Isolation of RNA and real-time RT-PCR

Total RNA was extracted from the liver and small intestine using the commercially available Trizol reagent (TRItidy G™, AppliChem, Germany). Following the DNase digestion, complementary DNAs (cDNAs) were synthesized from the samples using the commercially available synthesis kit, in respect to manufacturer’s description (Promega GmbH, USA). The genes listed in the Supplementary Table 1 were amplified using the real-time polymerase chain reaction, and the values were normalized to the 18S housekeeping gene values using the comparative delta delta Ct method ^81^.

### Microbiota analysis

DNA was extracted from small intestinal samples with FastDNATM Spin Kit for soil for metataxonomic analyses and quantified with NanoDrop 2000 spectrophotometer (Thermo Scientific, Waltham, USA). V1–V2 primers were used for 16S rRNA gene amplification^82^. Barcodes (6-nt) were attached to forward primers, and index adapters were linked to reverse primers. The library for sequencing was created with two rounds of PCR. In short, 1 µl of extracted DNA was used in the first PCR in a total of 20-µl volume, which also included 0.2 µl of PrimeSTAR HS DNA polymerase and 0.5 µl (0.2 μM) of each primer. The final product of the first PCR (1 µl) was then used for the second PCR in a total volume of 50 µl. The PCR started with denaturation at 95 °C for 3 min, followed by 15 cycles for the first PCR and 20 cycles for the second, denaturation at 98 °C (10 s) and subsequent annealing at 55 °C (10 s), elongation at 72 °C (45 s) and a final extension at 72 °C (2 min). After the second PCR, DNA concentration was normalized by the SequalPrep Normalization Kit (Invitrogen Inc., Carlsbad, USA). Sequencing was performed with the 250 bp paired-end Illumina NovaSeq 6000 platform.

### Statistical analysis

All values are presented as means ± standard error of the means (SEM). Statistical analyses were performed using PRISM software (version 7.03, GraphPad Software, Inc.). The Grubbs test was used to identify outliers. After testing for normal distribution, differences between the two groups were determined by unpaired students t-test, if data were normally distributed and non-parametric Mann-Whitney test was used, when data were not normally distributed. In case of >3 different groups a one-way ANOVA or two-way ANOVA was used. In case inhomogeneity of variance was detected, data were log-transformed. Differences were considered statistically significant if the *p*-value was <0.05.

For the analysis of data obtained when sequencing microbiota, sequencing fastq files were demultiplexed by Sabre (https://github.com/najoshi/sabre) and analyzed with Qiime2^83^. Primers and adapters were trimmed by the q2-cutadapt plugin^84^. The q2-dada2^85^ plugin was employed for denoising and merging paired reads, and chimeras removal. Resulted amplicon sequence variants (ASV) were annotated with VSEARCH-based consensus^86^ and pre-fitted sklearn-based classifiers^87^ against the Silva database (v138.1, 16S 99%)^88^. The reference sequences for the taxonomy assignment were obtained and preprocessed by RESCRIPt^89^. The estimation of alpha diversity was performed by Shannon’s entropy^90^ and Faith’s phylogenetic diversity (Faith’s PD) metrics^91^, and for beta diversity the robust Aitchison distances (RPCA)^92^ were calculated. Statistical analyses of alpha diversity indices were performed using the ANOVA test^93^ and beta diversity distances were compared using the Adonis test (999 permutations)^94^. The formula “Genotype + Cage” was used for both ANOVA and Adonis tests to account for the cage effect. Absolute counts of genera with relative abundance ≥1% and prevalence ≥10% were tested for differential abundance by the Ancom-BC test ^95^.

## Supplementary information

Supplemental Material

## Data Availability

The original contributions presented in the study are included in the article/ Supplementary Material and raw sequences were deposited to the European Nucleotide Archive (ENA) under accession number PRJEB76497. Further inquiries can be directed to the corresponding author.
