# Supplementary material for: TNFα is a trigger of aging-associated liver inflammation in mice
Source: NPJ Aging. 2026 Jan 13;12(1):27. doi: 10.1038/s41514-025-00326-w (PMC12894877; doi:10.1038/s41514-025-00326-w)
Supplement: Supplementary file 1 — Supplemental Material [file 41514_2025_326_MOESM1_ESM.pdf]

Supplemental Figures and Tables

TNFα is a Trigger of Aging-Associated Liver Inflammation in Mice

Haktan Övül Bozkir[1#], Annette Brandt[1#], Katja Csarmann[1], Anja Baumann[1], Katharina Burger[1], Timur Yergaliyev[2], Tim Hendrikx[3], Amélia Camarinha-Silva[2], Ina Bergheim[1]

# authors contributed equally

Corresponding author:
Ina Bergheim, PhD
University of Vienna
Department of Nutritional Sciences
R.F. Molecular Nutritional Science
Josef-Holaubek-Platz 2/ UZAII
A-1090 Vienna, Austria
Phone: +43 (1) 4277-54981
E-mail: ina.bergheim@univie.ac.at

Supplemental Table 1: Primer sequences used for real-time PCR.

	Forward (5´-3´)	*Reverse (5´-3´)*
18S	GTA ACC CGT TGA ACC CCA TT	CCA TCC AAT CGG TAG TAG CG
asma	CTG ACA GAG GCA CCA CTG AA	CAT CTC CAG AGT CCA GCA CA
Cd1d	TGT GTA CCA GTC CGG GAG CA	GTG CGG GAC CAG CTT CTG TT
Cd11b	TAC CGG AAG GAT TCA GCA AG	AAA GGA GGC ATG AGA GTC CA
Col1a1	ACG TGG AAA CCC GAG GTA TG	CTT GGG TCC CTC GAC TCC TA
Lgr5	GTG GTG GGA AGA CGG AAT CG	AAT GGT CAG GGC CAA CAG GA
Mcp1	GGG CCT GCT GTT CAC AGT T	CCA GCC TAC TCA TTG GGA T
Mmp2	TGT GCC ACC ACC GAG GAC TA	GCA CAC CAC ACC TTG CCA TC
Nk1.1	GTG TCT TAG TGC GAG TCT TAG	GGG CAC TCT AAA TTA ACT GAA
p16	CCA AGA GCG GGG ACA TCA AG	AAG AAA AAG GCG GGC TGA GG
p21	GCC CCT CTG ACC TGC ACT GG	AGG GAC AGC AGC AGA GGG GA
Tert	ACC TGC CGA CCT TTC CTT CC	GCC TCT GGC CTC GTT AAG CA
Timp2	CTG GCC CTT HCA AAT GCT TC	GCT CAG GGA GGG CTT CCA TT
Tlr4	AGC CAT TGC TGC CAA CAT CA	GCT GCC TCA GCA GGA CTT C
Tnfa	CAG CCA ACC AGG CAG GTT CT	CCT GCC ACA AGC AGG AAT GA

asma - α smooth muscle actin, Col1a1 - collagen type I α 1 chain, Lgr5 - Leucine-rich repeat-containing G-protein coupled receptor 5, Mcp1 - Monocyte chemoattractant protein-1, Mmp2 - Matrix metalloproteinase 2, Tert - telomerase reverse transcriptase, Timp2 - tissue inhibitor of metalloproteinase 2, Tlr4 - toll-like receptor 4, Tnfa – Tumor necrosis factor α

The ARRIVE Essential 10: Compliance Questionnaire

Use this questionnaire to evaluate how well a manuscript complies with the ARRIVE Essential 10. It can be applied to any manuscript describing comparative experiments in living animals, by assessors such as journal staff, editors, or peer reviewers.

Item		Question(s)	Answers
1	Study Design	Are all experimental and control groups clearly identified?	☒ Yes, for at least one experiment<br>☐ No
		Is the experimental unit (e.g. an animal, litter or cage of animals) clearly identified?	☒ Yes, for at least one experiment<br>☐ No
2	Sample Size	Is the exact number of experimental units in each group at the start of the study provided (e.g. in the format '*n*=')?	☒ Yes, for at least one experiment<br>☐ No
		Is the method by which the sample size was chosen explained?	☒ Yes, for at least one experiment<br>☐ No
3	Inclusion & Exclusion Criteria	Are the criteria used for including and excluding animals, experimental units, or data points provided?	☒ Yes, for at least one experiment<br>☐ No
		Are any exclusions of animals, experimental units, or data points reported, or is there a statement indicating that there were no exclusions?	☒ Yes, for at least one analysis<br>☐ No
4	Randomisation	Is the method by which experimental units were allocated to control and treatment groups described?	☒ Yes, for at least one experiment<br>☐ No
5	Blinding	Is it clear whether researchers were aware of, or blinded to, the group allocation at any stage of the experiment or data analysis?	☒ Yes, for at least one experiment<br>☐ No
6	Outcome Measures	For all experimental outcomes presented, are details provided of exactly what parameter was measured?	☒ Yes, for at least one experiment<br>☐ No
7	Statistical Methods	Is the statistical approach used to analyse each outcome detailed?	☒ Yes, for at least one analysis<br>☐ No
		Is there a description of any methods used to assess whether data met statistical assumptions?	☒ Yes, for at least one analysis<br>☐ No<br>☐ Not applicable
8	Experimental Animals	Are all species of animal used specified?	☒ Yes, for at least one experiment<br>☐ No
		Is the sex of the animals specified?	☒ Yes, for at least one experiment<br>☐ No<br>☐ Not applicable to species
		Is at least one of age, weight or developmental stage of the animals specified?	☒ Yes, for at least one experiment<br>☐ No
9	Experimental Procedures	Are both the timing and frequency with which procedures took place specified?	☒ Yes, for at least one experiment<br>☐ No
		Are details of acclimatisation periods to experimental locations provided?	☒ Yes, for at least one experiment<br>☐ No
10	Results	Are descriptive statistics for each experimental group provided, with a measure of variability (e.g. mean and SD, or median and range)?	☒ Yes, for at least one experiment<br>☐ No<br>☐ Not applicable to the type of data collected
		Is the effect size and confidence interval provided?	☐ Yes, for at least one experiment<br>☐ No<br>☒ Not applicable to the type of analysis used

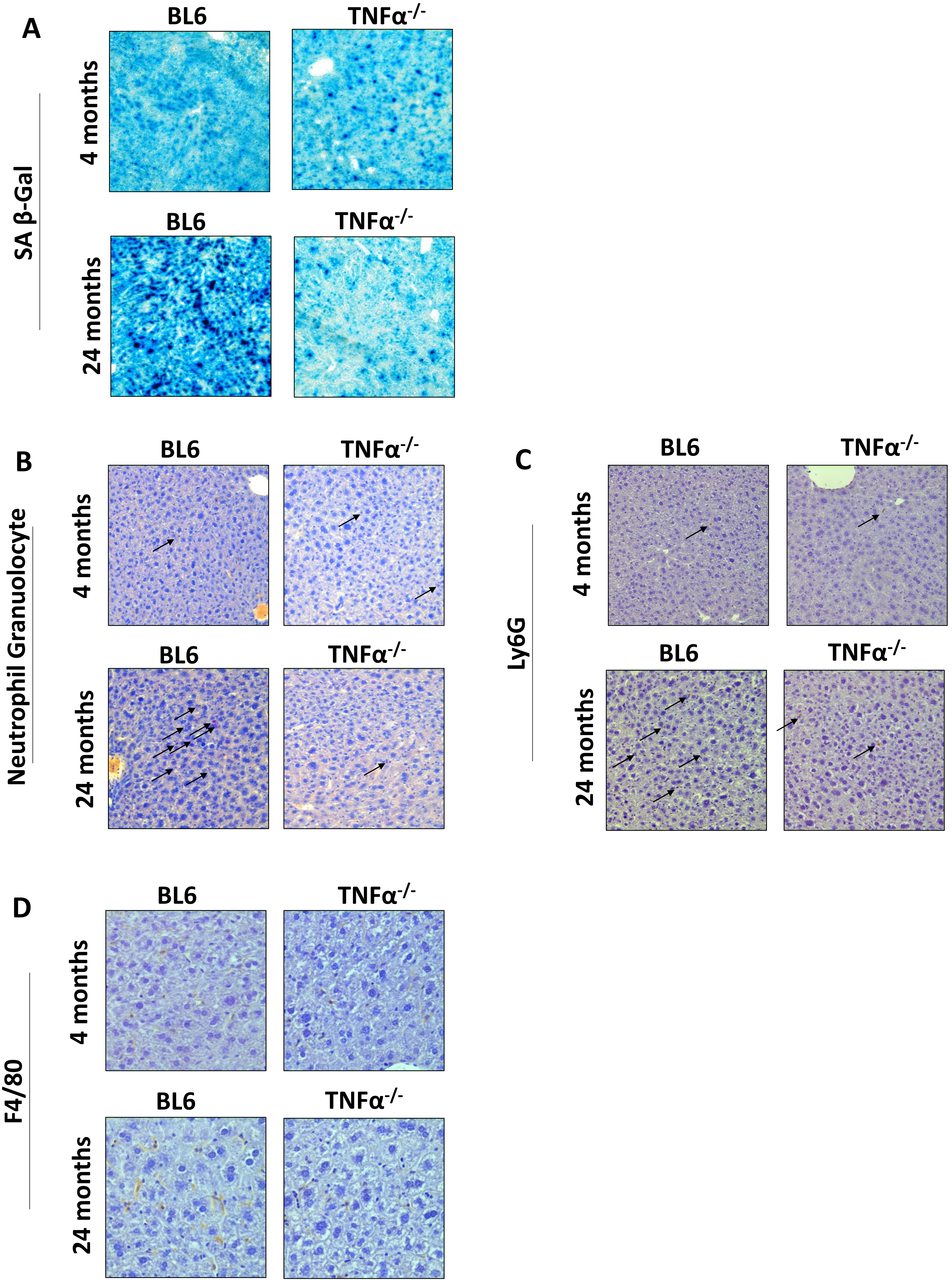

Supplemental Figure 1: Representative pictures of immunohistochemical stainings in liver sections from Figure 2. Representative pictures of staining of (A) Senescence-associated β-Galactosidase (SA β-Gal) (200x), (B) neutrophil granulocytes (200x), (C) Lymphocyte antigen 6 complex locus G6D (Ly6G) positive cells (200x) and (D) F4/80 positive cells (400x) in liver sections. B-C: Arrows indicate positive stained cells.

Blots Figure 3H

Merged with ladder | **Bands only**

A

Occludin

37kDa

25kDa

TNFα (10ng/ml) - +

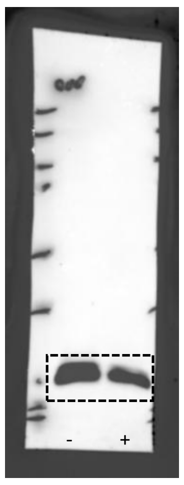

TNFα (10ng/ml) - +

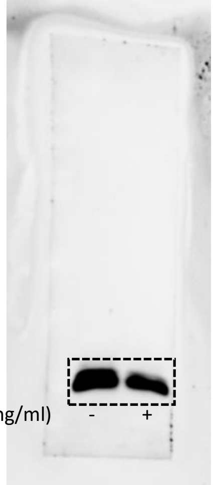

Occludin
25kDa

B

β-actin

50kDa

37kDa

TNFα (10ng/ml) - +

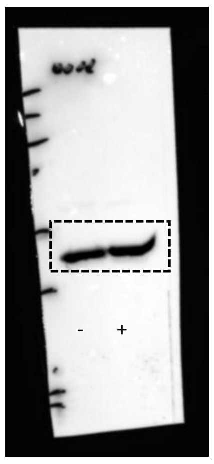

TNFα (10ng/ml) - +

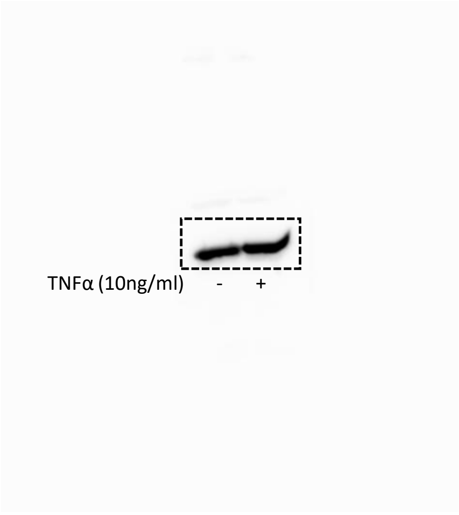

β-actin
43kDa

Supplemental Figure 2: Original blots of Figure 3H. Original blots of (A) Occludin and (B) β-actin blots of Figure 3H. Left side: Bands merged with ladder, Right side: Bands only. Bands that are shown in Figure 3H are highlighted with a frame.

Blots Figure 3J

A

Merged with ladder

Occludin

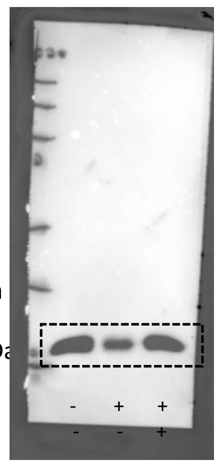

Bands only

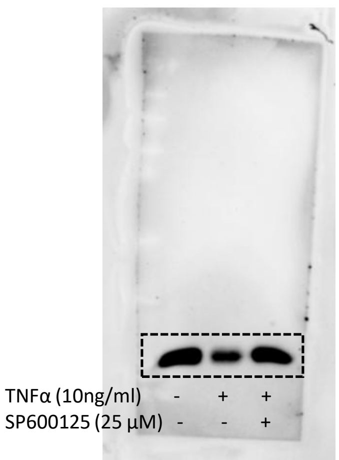

Occludin 25kDa

B

β-actin

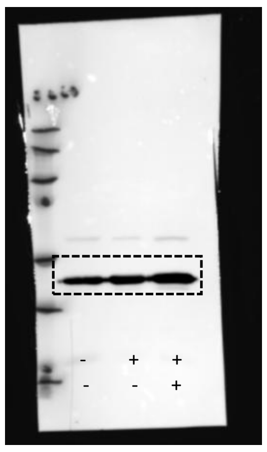

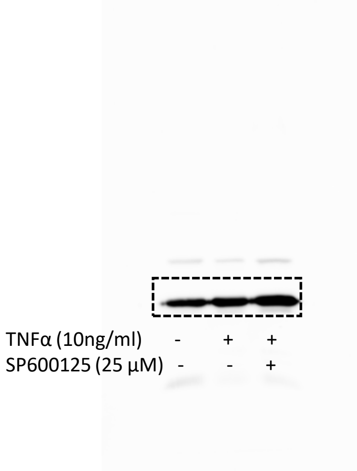

β-actin 43kDa

Supplemental Figure 3: Original blots of Figure 3J. Original blots of (A) Occludin and (B) β-actin blots of Figure 3J. Left side: Bands merged with ladder, Right side: Bands only. Bands that are shown in Figure 3J are highlighted with a frame.

Blots Figure 3K

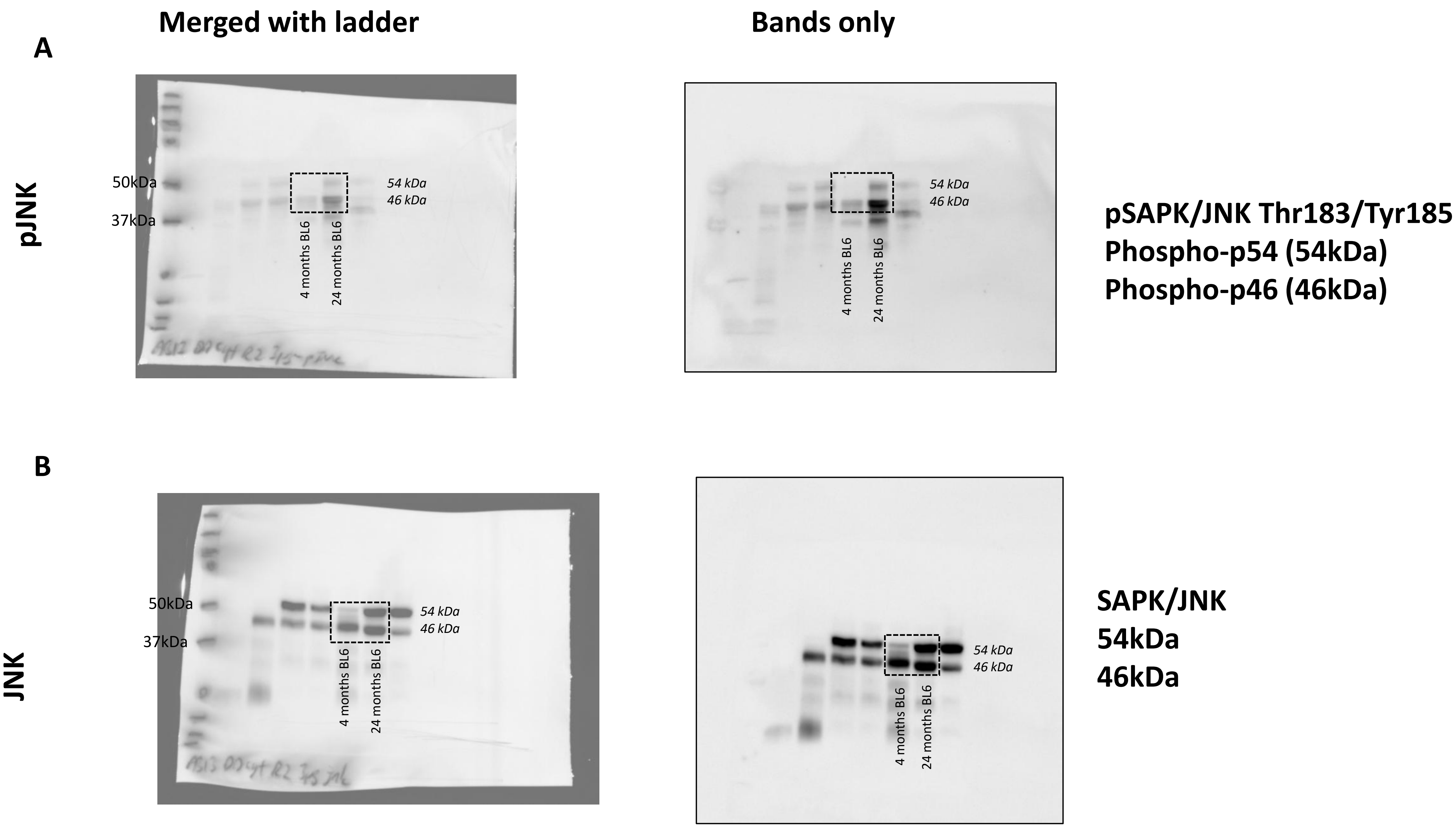

Supplemental Figure 4: Original blots of Figure 3K. Original blots of (A) phospho c-jun N-terminal kinases (pJNK) and (B) JNK blots of Figure 3K. Left side: Bands merged with ladder, Right side: Bands only. Bands that are shown in Figure 3K are highlighted with a frame.
